# Differences in Ambient Polycyclic Aromatic Hydrocarbon Concentrations between Streets and Alleys in New York City: Open Space *vs.* Semi-Closed Space

**DOI:** 10.3390/ijerph13010127

**Published:** 2016-01-12

**Authors:** Stephanie Lovinsky-Desir, Rachel L. Miller, Joshua Bautista, Eric N. Gil, Steven N. Chillrud, Beizhan Yan, David Camann, Frederica P. Perera, Kyung Hwa Jung

**Affiliations:** 1 Division of Pulmonology, Department of Pediatrics, College of Physicians and Surgeons, Columbia University, 3959 Broadway, CHC 7-724, New York, NY 10032, USA; sl3230@cumc.columbia.edu; 2 Division of Pulmonary, Allergy and Critical Care of Medicine, Department of Medicine, College of Physicians and Surgeons, Columbia University, PH8E-101, 630 W. 168 St., New York, NY 10032, USA; rlm14@cumc.columbia.edu (R.L.M.); jbb2160@cumc.columbia.edu (J.B.); ericngil3@gmail.com (E.N.G.); 3 Department of Environmental Health Sciences, Mailman School of Public Health, Columbia University, 722 W. 168 St. New York, NY 10032, USA; fpp1@columbia.edu; 4 Division of Pediatric Allergy and Immunology, Department of Pediatrics, College of Physicians and Surgeons, Columbia University, PH8E-101, 630 W. 168 St. New York, NY 10032, USA; 5 Lamont-Doherty Earth Observatory, Columbia University, 61 Rt., 9W Palisades, New York, NY 10964, USA; chilli@ldeo.columbia.edu (S.N.C.); by2142@columbia.edu (B.Y.); 6 Chemistry and Chemical Engineering Division, Southwest Research Institute, 6220 Culebra Road, San Antonio, TX 78228, USA; david.camann@swri.org

**Keywords:** ambient polycyclic aromatic hydrocarbons, spatial variation, alley *vs*. street, aged air, open *vs.* semi-closed space, built environment

## Abstract

*Background:* Outdoor ambient polycyclic aromatic hydrocarbon (PAH) concentrations are variable throughout an urban environment. However, little is known about how variation in semivolatile and nonvolatile PAHs related to the built environment (open space *vs.* semi-closed space) contributes to differences in concentrations. *Methods:* We simultaneously collected 14, two-week samples of PAHs from the outside of windows facing the front (adjacent to the street) open side of a New York City apartment building and the alley, semi-closed side of the same apartment unit between 2007 and 2012. We also analyzed samples of PAHs measured from 35 homes across Northern Manhattan and the Bronx, 17 from street facing windows with a median floor level of 4 (range 2–26) and 18 from alley-facing windows with a median floor level of 4 (range 1–15). *Results:* Levels of nonvolatile ambient PAHs were significantly higher when measured from a window adjacent to a street (an open space), compared to a window 30 feet away, adjacent to an alley (a semi-closed space) (street geometric mean (GM) 1.32 ng/m^3^, arithmetic mean ± standard deviation (AM ± SD) 1.61 ± 1.04 ng/m^3^; alley GM 1.10 ng/m^3^, AM ± SD 1.37 ± 0.94 ng/m^3^). In the neighborhood-wide comparison, nonvolatile PAHs were also significantly higher when measured adjacent to streets compared with adjacent to alley sides of apartment buildings (street GM 1.10 ng/m^3^, AM ± SD 1.46 ± 1.24 ng/m^3^; alley GM 0.61 ng/m^3^, AM ± SD 0.81 ± 0.80 ng/m^3^), but not semivolatile PAHs. *Conclusions:* Ambient PAHs, nonvolatile PAHs in particular, are significantly higher when measured from a window adjacent to a street compared to a window adjacent to an alley, despite both locations being relatively close to street traffic. This study highlights small-scale spatial variations in ambient PAH concentrations that may be related to the built environment (open space *vs.* semi-closed space) from which the samples are measured, as well as the relative distance from street traffic, that could impact accurate personal exposure assessments.

## 1. Introduction

Polycyclic aromatic hydrocarbons (PAHs) are naturally occurring combustion byproducts of coal, petroleum, and gasoline. PAHs are emitted readily into the environment and have been associated with negative health outcomes including respiratory disease [1,2,3,4], cardiovascular disease [1,5] and cancer [5,6,7]. Of particular concern in urban environments is exposure to PAHs from pervasive outdoor sources including automobile, diesel fuel, and heating oil emissions [8,9]. A better understanding of how exposure to PAHs may vary by location, specifically within short distances from dominant sources, is critical to informing PAH exposure models based on locations of and within apartment buildings in urban environments.

Important differences in spatial distribution of outdoor ambient PAHs have been described [10,11,12]. For example, Jaward *et al*. reported large-scale PAH spatial variations in air samples collected on a ship traveling from The Netherlands to South Africa [12]. Lee *et al.* also reported highest PAH concentrations measured at a major intersection with heavy traffic, followed by urban and rural locations in Taiwan [11]. Within a city concentrations of outdoor airborne PAHs can also vary significantly [13,14,15]. For example, Nielsen described higher ambient PAH concentrations measured along a busy street in Copenhagen compared to concentrations measured in a park, several meters away [13]. Similarly, a recent study observed a clear horizontal concentration gradient of PAHs within 150 meters of a highway in New Jersey [16]. Our group also described a vertical gradient in ambient PAH concentrations whereby the concentrations of outdoor PAHs measured from an apartment building window on the 6th floor or higher were lower than those measured at lower floors [17]. These findings can be explained by differences in proximity to roadways and traffic emission sources on a relatively large scale. However, variability of PAH concentrations in different structural environments (e.g., open space *vs.* semi-closed spaces) within short distances from traffic emission sources, have not been well-studied.

PAHs have two types of anthropogenic sources: pyrogenic (*i.e.*, incomplete combustion of organic materials such as traffic emissions and heating oil) and petrogenic (*i.e.*, unburned fossil organic materials such as direct evaporation from petroleum products). Petrogenic *vs.* pyrogenic emission ratios of semivolatile (*i.e.*, low molecular weight) *vs.* nonvolatile (*i.e.*, high molecular weight) PAHs often differ [18]. For example, methylphenanthrenes, semivolatile PAHs, are emitted more abundantly from petrogenic sources [19], whereas the predominant source of nonvolatile PAHs is from traffic emissions, a pyrogenic source [20,21,22]. In addition, studies support a distinction between semivolatile and nonvolatile PAHs in atmospheric behaviors [20,21,22] and by season [23] as well as exposure-related health outcomes. For example, we reported asthma was linked to exposure to semivolatile PAHs [4] while obesity was linked to exposure to nonvolatile PAHs [24]. Therefore, a greater understanding of the spatial characteristics of PAHs relies on considerations of nonvolatile *vs.* semivolatile PAHs concentrations.

It is well known that ambient PAHs from predominately outdoor sources often vary across different rooms within homes [25,26,27]. However, it is unknown if PAH concentrations measured from a window at the front of a building (in open space, adjacent to a street) varies from concentrations measured from a window at the side or back of a building (semi-closed space, in an alley), limiting accurate personal exposure assessments. Therefore, our objective was to characterize nonvolatile and semivolatile outdoor ambient PAHs measured at the front, open side compared with alley, semi-closed side of a building in an urban neighborhood in New York City (NYC). We hypothesized that even across a short distance relative to street traffic, the dominant emission source, there would be a difference in PAH concentrations, especially the nonvolatile PAHs. In addition, we hypothesized that the difference in PAH concentrations would remain apparent between frontage *vs.* alley sides of buildings throughout the urban NYC neighborhood.

## 2. Materials and Methods

### 2.1. Central Site Outdoor Air Monitoring

Ambient outdoor levels of PAH were measured once each season from two different locations within one central site, approximately 9 m apart, from October 2007 to May 2012 as a part of quality assurance/quality control (QAQC). Two week integrated air monitoring was collected at a flow rate of 1.5% ± 15% L/min, with an average volume of 30.1 m^3^ [21]. Particulate phase of PAH was collected on a quartz microfiber filter in a cassette attached downstream from a cyclone with a 2.5 µm aerodynamic-diameter cut point (model SCC 1.062, BGI, Inc., Butler, NJ, USA). Gas phases of PAH were collected on polyurethane foam (PUF) cartridge back-up, as previously described [28].

**Figure 1:**
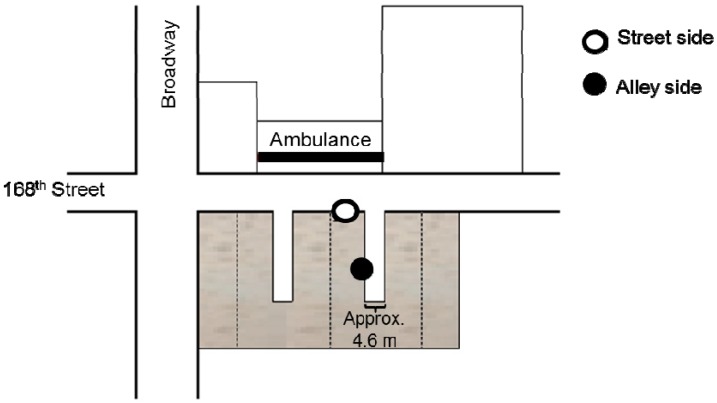
Central site monitor placement: street *vs.* alley side windows of the same NYC apartment unit. Note: The alley (●) monitor was nine meters away from the street monitor (○) and approximately 4.6 meters away from adjacent building.

The central site was located on the 5th floor of a six story apartment building in NYC. The “street” monitor was positioned outside the front window of the apartment unit, located across the street from a highly active ambulance bay where diesel trucks often idle (Figure 1). Data were collected from the street monitor once per meteorological season in two week blocks. The “alley” monitor was positioned outside the back window of the same apartment unit (nine meters from the street monitor), adjacent to another building approximately 4.6 meters away (Figure 1). Data were collected from the alley monitor continuously throughout the study period in two week blocks. The street and alley measurement blocks were simultaneous for quality control/quality assurance.

### 2.2. Residential Outdoor Air Monitoring

Non-smoking Dominican and African American women ages 18–35 years residing in Northern Manhattan and the South Bronx were enrolled during pregnancy in the Columbia Center for Children’s Environmental Health (CCCEH) birth cohort and their children were followed prospectively. Outdoor measurement of PAH levels were collected from the children’s homes at age 9–11 years for two weeks employing the same methodology used at the central site. Monitors were placed outside a window in the room the child spent the majority of her or his time. Detailed home and traffic exposure assessment was conducted by the research staff to document the window site of outdoor monitor placement, apartment floor level, total building floor height, and number of lanes on the street of residence. For the purposes of this study, among the *n* = 56 homes with outdoor PAH measurements, we performed analysis on the 35 homes for which home and traffic exposure assessment data were available. Street was defined as window facing “street of residence” (*n* = 12) or “other street” (*n* = 5). Alley was defined as a window facing the “side of building” (*n* = 7), “inner courtyard” (*n* = 7), or “back of building” (*n* = 4). The 17 street facing window measurements were from a median apartment floor level of 4 (range 2–26) and 18 alley facing window measurements also from a median apartment floor level of 4 (range 1–15) (Table 1).

**Table 1:** Building environment.

Location of Monitor Placement	Number of Measurements	Building Floor Height	Number of Lanes on Street of Address
Central Site Street	14	5	2
Central Site Alley	14	5	2
Neighborhood Streets	17	4 (2–26) ^**a**^	2 (1–4) ^**a**^
Neighborhood Alleys	18	4 (1–15) ^**a**^	2 (1–4) ^**a**^

**^a^** Median (minimum–maximum).

### 2.3. Polycyclic Aromatic Hydrocarbon

Sixteen four-ring to six-ring PAHs were selected as target compounds due to their abundance in traffic emissions and their possible carcinogenicity and mutagenicity [29,30]. Sixteen PAHs were monitored: benz[a]anthracene (BaA), chrysene/iso-chrysene (Chry), benzo[b]fluoranthene (BbFA), benzo[k]fluoranthene (BkFA), benzo[a]pyrene (BaP), indeno[1,2,3-c,d]pyrene (IP), dibenz[a,h]anthracene (DahA), benzo[g,h,i]perylene (BghiP), pyrene (Pye), phenanthrene (Phe), 1-methylphenanthrene (1Meph), 2-methylphenanthrene (2Meph), 3-methylphenanthrene (3Meph), 9-methylphenanthrene (9Meph), 1,7-dimethylphenanthrene (1,7DMeph), and 3,6-dimethylphenanthrene (3,6DMeph). A single soxhlet extraction of both the filters and PUFs together were analyzed for total (gas + particulate) PAHs at Southwest Research Institute as previously described [21,31]. Two deuterated compounds (anthracene-d10 and p-terphenyl-d14) were used as surrogate standards for recovery and chrysene-d12 and perylene-d12 were used as internal standards for quantification [21]. The limit of detection (LOD) for the 15 PAHs and phenanthrene was 1 ng and 4.5 ng, respectively. All PAHs were above LODs, with the exception of BkFA (80.2% above LOD), BaP (80.2% above LOD) and DaAh (37.5% above LOD).We replaced observations below the LOD with half the detection limit and further converted to air concentration (ng/m³) based on air volume collected (m³).

### 2.4. Data Analysis

PAH levels were summed according to their relative volatility and gas/particle partitioning *i.e.*, Σ_8_PAH_semivolatile_ (sum of eight low molecular-weight-PAH ≤ 206) and Σ_8_PAH_nonvolatile_ (sum of eight high molecular-weight-PAH ≥ 228). Heating season was defined as any sampling that was initiated 1 October through 30 April as described [21]. Nonparametric tests were performed due to the non-normal distribution of individual PAHs. Spearman’s rho was used to determine rank-order correlations and Wilcoxon signed rank test was used for comparisons of paired samples. Data analysis was conducted with SPSS version 22.0 (SPSS Inc., Chicago, IL, USA). All tests were two-sided with significance level of 0.05.

## 3. Results

### 3.1. Central Site Street vs. Alley PAH Concentrations

**Figure 2:**
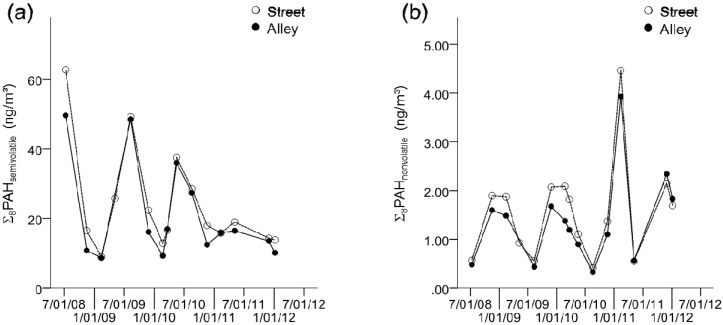
Central site comparison of (**a**) Σ_8_PAH_semivolatile_ and (**b**) Σ_8_PAH_nonvolatile_ concentrations: street *vs.* alley. Notes: Each dot represents one measurement collected over two weeks. (**a**) the sum of eight semivolatile PAHs (Σ_8_PAH_semivolatile_: Phe, 1Meph, 2Meph, 3Meph, 9Meph, 1,7DMeph, 3,6DMeph, and Pyrene); (**b**) the sum of eight nonvolatile PAHs (Σ_8_PAH_nonvolatile_: BaA, Chry, BbFA, BkFA, BaP, IP, DahA, and BghiP). The white circle represents measurements from the street side monitor and the black circle from the alley side monitor.

A total of 14 outdoor ambient PAH samples, representing all four meteorological seasons, were collected simultaneously from the front of the building, adjacent to the street and at the back of the building, adjacent to the alley of the same NYC apartment unit. Geometric mean concentrations of Σ_8_PAH_nonvolatile_ measured from the street side were 18% higher than those measured from the alley side of the central site building (1.32 ng/m^3^ street side *vs*. 1.10 ng/m^3^ alley side). Nearly all of the individual nonvolatile PAHs were higher at the street side with the greatest relative differences in Daha (40%), followed by BghiP (32%), and BbFA (25%) (Figure 2 and Table 2, Wilcoxon signed rank test, *p* < 0.05).

**Table 2:** Comparison of central site PAH concentrations between street and alley sides of the building for *n* = 14 two-week sampling periods.

Compounds	Street	Alley	Relative Difference ^a^ (%)	Wilcoxon *p*-Value ^b^	Correlation Coefficient ^c^
Σ_8_PAH_semivolatile_	20.59 (24.00 ± 15.49)	17.46 (20.81 ± 14.04)	16	0.002	0.93 ***
Pyrene	0.89 (0.94 ± 0.34)	0.83 (0.89 ± 0.33)	7	0.198	0.90 ***
Phe	15.50 (18.32 ± 12.35)	12.63 (15.40 ± 10.98)	21	0.001	0.94 ***
1-MEPH	0.58 (0.68 ± 0.41)	0.55 (0.64 ± 0.41)	5	0.109	0.94 ***
2-MEPH	1.14 (1.32 ± 0.80)	1.07 (1.24 ± 0.78)	6	0.064	0.90 ***
3-MEPH	1.27 (1.47 ± 0.93)	1.18 (1.39 ± 0.90)	7	0.084	0.94 ***
9-MEPH	0.81 (0.94 ± 0.59)	0.78 (0.93 ± 0.62)	4	0.363	0.96 ***
1,7-DMEPH	0.10 (0.13 ± 0.09)	0.10 (0.12 ± 0.09)	0	0.975	0.81 ***
3,6-DMEPH	0.18 (0.21 ± 0.11)	0.18 (0.20 ± 0.11)	0	0.470	0.82 ***
Σ_8_PAH_nonvolatile_	1.32 (1.61 ± 1.04)	1.10 (1.37 ± 0.94)	18	0.011	0.90 ***
BaA	0.08 (0.10 ± 0.07)	0.08 (0.10 ± 0.07)	0	0.363	0.89 ***
Chry	0.16 (0.19 ± 0.11)	0.15 (0.18 ± 0.11)	6	0.683	0.93 ***
BbFA	0.31 (0.40 ± 0.26)	0.24 (0.31 ± 0.21)	25	0.002	0.83 ***
BkFA	0.07 (0.10 ± 0.07)	0.06 (0.08 ± 0.05)	15	0.064	0.90 ***
BaP	0.07 (0.09 ± 0.08)	0.07 (0.10 ± 0.09)	0	0.470	0.93 ***
BghiP	0.40 (0.48 ± 0.32)	0.29 (0.37 ± 0.27)	32	0.004	0.91 ***
IP	0.19 (0.23 ± 0.16)	0.18 (0.22 ± 0.15)	5	0.056	0.97 ***
Daha	0.03 (0.03 ± 0.02)	0.02 (0.02 ± 0.01)	40	0.019	0.61 ***

N = 14; Geometric mean concentrations (Arithmetic mean ± standard deviation) presented. Units expressed in ng/m^3^ for PAH. **^a^** Difference of geometric PAH concentrations between street and alley, divided by average (%); **^b^** Wilcoxon rank sum test performed; **^c^** Spearman correlation coefficients; *** *p*-value < 0.001 for all correlations between alley and street. Σ_8_PAH_semivolatile_: Phe, 1Meph, 2Meph, 3Meph, 9Meph, 1,7DMeph, 3,6DMeph, and Pyrene. Σ_8_PAH_nonvolatile_: BaA, Chry, BbFA, BkFA, BaP, IP, DahA, and BghiP.

A similar pattern was observed for Σ_8_PAH_semivolatile_ with 16% higher geometric mean concentrations measured at the street side compared to the alley side of the central site building (20.59 ng/m^3^ street side *vs* 17.46 ng/m^3^ alley side). However, this difference was driven mainly by phenanthrene (21% difference; 15.50 ng/m^3^ street side *vs* 12.63 ng/m^3^ alley side). The other individual semivolatile PAHs were not significantly different between street and alley sides (Figure 2 and Table 2, Wilcoxon signed rank test, *p* > 0.05).

Despite the observed differences, both Σ_8_PAH_semivolatile_ and Σ_8_PAH_nonvolatile_ measured at the street side were highly correlated with those measured at the alley side (Table 2. Spearman correlation, Σ_8_PAH_semivolatile_ r = 0.93, *p* ≤ 0.001; Σ_8_PAH_nonvolatile_ r = 0.90, *p* ≤ 0.001). A similar trend was observed for all individual 16 PAH (Spearman 0.61 ≤ r ≤ 0.94; *p* < 0.001).

### 3.2. Neighborhood-Wide Alley vs. Street PAH Concentrations

A total of 35 residential outdoor ambient PAH monitors were interrogated throughout Northern Manhattan and the Bronx in NYC; 17 of which were placed outside of a window facing a street and 18 outside of a window facing an alley. The building characteristics of the neighborhood-wide sample were comparable to the central site including a median floor height of four (range 1–26) and an average of two lanes on the street adjacent to the building (Table 1).

**Figure 3:**
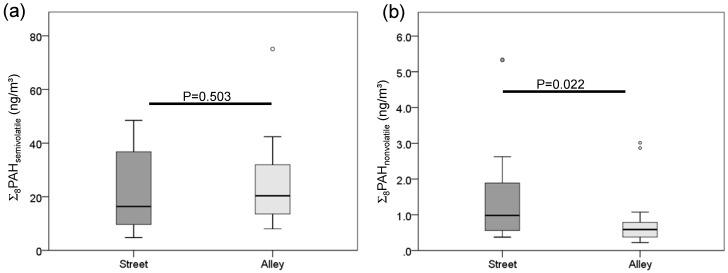
Neighborhood-wide comparison of residential outdoor (**a**) Σ_8_PAH_semivolatile_ and (**b**) Σ_8_PAH_nonvolatile_ levels in NYC: street *vs.* alley. Note: Geometric mean concentrations and 95% confidence intervals presented. Mann-Whitney test performed to compare PAH concentrations measured from the street side of buildings *vs.* the alley side of buildings across the neighborhood wide sample.

The geometric mean Σ_8_PAH_nonvolatile_ concentration measured from the street side of buildings was 57% higher than that from the alley side of buildings across the neighborhood wide sample (Figure 3 and Table 3. 1.10 ng/m^3^ street side *vs*. 0.61 ng/m^3^ alley side; Mann-Whitney test, *p* = 0.022). A similar trend was observed for individual nonvolatile PAHs, except for DahA and this pattern was more apparent during the heating season, compared to nonheating season (Table 3). Unlike the nonvolatile PAHs, neither Σ_8_PAH_semivolatile_ nor individual semivolatile PAH concentrations were significantly different between alley and street locations.

**Table 3:** NYC neighborhood-wide comparison of PAHs measured at street and alley sides of buildings; stratified by heating season.

Compounds	Overall				Heating Season ^b^			Nonheating Season ^b^		
	Street (*n* = 17)	Alley (*n* = 18)	Relative Difference ^a^ (%)	*p*	Street (*n* = 11)	Alley (*n* = 7)	Relative Difference ^a^ (%)	Street (*n* = 6)	Alley (*n* = 11)	Relative Difference ^a^ (%)
**Σ**_8_**PAH_semivolatile_**	17.4	21.3	−20	0.503	12.3	12.0	2	32.6	30.7	6
Pyrene	0.92	0.82	11	0.258	0.80	0.62	25	1.16	0.97	18
Phe	12.0	15.5	−25	0.424	8.26	8.47	−3	23.9	22.9	5
1-MEPH	0.58	0.67	−14	0.568	0.41	0.39	5	1.09	0.95	14
2-MEPH	1.13	1.34	−17	0.568	0.83	0.82	1	2.01	1.83	9
3-MEPH	1.22	1.41	−14	0.590	0.88	0.86	2	2.22	1.94	13
9-MEPH	0.92	0.97	−5	0.708	0.69	0.58	17	1.57	1.34	16
1,7-DMEPH	0.10	0.11	10	0.405	0.07	0.05	33	0.19	0.19	0
3,6-DMEPH	0.22	0.23	−4	0.757	0.17	0.13	27	0.38	0.33	14
**Σ**_8_**PAH**_**nonvolatile**_	1.10	0.61	57	**0.022**	1.66	1.15	36	0.52	0.41	24
BaA	0.08	0.05	46	**0.038**	0.11	0.08	32	0.04	0.04	0
Chry	0.12	0.07	53	**0.025**	0.18	0.12	40	0.06	0.06	0
BbFA	0.24	0.12	67	**0.019**	0.39	0.24	48	0.10	0.07	35
BkFA	0.05	0.03	50	**0.041**	0.10	0.05	67	0.02	0.02	0
BaP	0.07	0.04	55	0.077	0.11	0.08	32	0.03	0.03	0
BghiP	0.33	0.18	59	**0.032**	0.48	0.37	26	0.16	0.11	37
IP	0.17	0.09	62	**0.029**	0.25	0.18	33	0.08	0.06	29
Daha	0.02	0.02	0	0.858	0.02	0.02	0	0.02	0.02	0

Geometric mean concentrations (Arithmetic mean ± standard deviation) presented. Units expressed in ng/m^3^ for PAH. Mann-Whitney test performed. **^a^** Difference of geometric PAH concentrations between street and alley, divided by average (%). **^b^** Heating season (October–April) and nonheating season (May–September). Σ_8_PAH_semivolatile_: Phe, 1Meph, 2Meph, 3Meph, 9Meph, 1,7DMeph, 3,6DMeph, and Pyrene. Σ_8_PAH_nonvolatile_: BaA, Chry, BbFA, BkFA, BaP, IP, DahA, and BghiP.

## 4. Discussion

Outdoor ambient PAH concentrations are variable throughout NYC. Here we have illustrated that differences exist in concentrations measured in open *vs.* semi-closed spaces, even when both locations are within short distance relative to traffic emission sources and outside the same apartment. In our sample of 14 simultaneous monitoring periods, nonvolatile ambient PAHs were significantly higher when measured from a window adjacent to a street, closer to traffic emissions (*i.e.*, open space), compared to a window nine meters away, adjacent to an alley (*i.e.*, semi-closed space). Furthermore, in a neighborhood-wide comparison, nonvolatile PAHs were also significantly higher when measured at the street sides compared with the alley sides of apartment buildings, but not semivolatile PAHs. This study highlights small-scale spatial variations in ambient PAH concentrations that may be related to the structural built environment from which the samples are measured as well as the relative distance from street traffic and may influence personal exposure.

In an effort to understand differences in PAH concentrations related to distance from traffic sources, Nielsen investigated airborne PAHs along a busy street and a few hundred meters away in an adjacent park in Copenhagen, Denmark [13]. Both nonvolatile and semivolatile PAHs were noted to be higher along the street, closer to traffic emission sources. Our central site findings on the micro level are consistent with those of Nielsen on the macro level, in that PAHs measured closest to street traffic were higher than concentrations measured only about nine meters away at the alley side of the same apartment building. Our findings were more robust for the nonvolatile PAHs which are predominately derived from pyrogenic sources such as traffic and heating oil emissions compared with the semivolatile PAHs [20,21,22]. Thus, we support the work of others that have described horizontal concentration gradient of PAHs associated with increasing distances from major roadways due to dilution effect [16,32]. While prior studies have focused on measurement of the dilution gradient at ground level, our measurements were taken from the 5th story apartment window. The height of the building also may influence the dilution effect since the air mass travels up and over the building from ground level emissions, given our previously published evidence on vertical gradients in PAH concentrations [17]. Our study further illustrates the dilution effect is maintained even above ground level, especially for nonvolatile PAHs whose major sources were traffic emissions at our central site.

In addition, differences in the structural environment surrounding the two sampling locations at our central site may influence the differences in PAHs between the two locations. The street side monitor was placed in an open location at the front of the building, directly adjacent to an active ambulance bay with idling vehicles, while the alley provided a semi-closed space surrounded by other buildings. Lower PAH concentrations from the alley could be due to the possibility that air sampled from the alley was more stagnant because of limited fresh air exchange, thus rendering the PAHs more subject to losses (particle impaction or degradation) compared with the more frequently circulated air sampled from the front, or street side of the building. Unlike other pollutants, such as elemental carbon or metal components, PAHs, in particular nonvolatile PAHs, have been shown to degrade during transport and deposition. This degradation can differ by the volatility, photo-transformation rate and bio-degradation rate of individual PAHs [33,34]. In addition, there is a growing body of literature to addresses concerns that “urban street canyons” (streets that are flanked on both sides by tall buildings) have some of the highest concentrations of traffic related particulate pollutants [35,36,37]. Ng and Chau used mathematical models to calculate the potential exposure to pollutants in different micro-environments within urban street canyon and demonstrated that building setbacks, or distance from the road, reduced personal exposure [38]. Therefore, another potential interpretation of our results is that the structural environment of the alley side of the building offers protection from the urban street canyon effect of exposure to pollutants, similar to that of a building setback.

In our neighborhood-wide comparison, we observed a consistent pattern in that the levels of Σ_8_PAH_nonvolatile_ as well as most of the individual nonvolatile PAHs measured from the street side of buildings were also higher than those from alleys. This pattern was more apparent during the heating season, compared to the nonheating season. However, we did not observe appreciable differences in Σ_8_PAH_semivolatile_. Our lack in significant difference in neighborhood-wide semivolatile PAHs may be related to seasonal variation, which is known to be one of the most important factors affecting ambient PAH concentrations [21]. For example, semivolatile PAH concentrations are substantially higher during the nonheating season compared to heating season [21]. Unlike the central site paired comparison between alley and street, most samples from the neighborhood-wide comparison were not concurrent. The lack of appreciable difference between neighborhood-wide street and alley Σ_8_PAH_semivolatile_ may be explained by uneven sample collection from either season (Table 3. eleven samples in nonheating season, seven samples in heating season), canceling out the effect of the built environment. This was supported by stratified analysis by heating season (Table 3). Although our small sample size limited our power to detect significant differences, after controlling for heating season, there was a trend toward higher street *vs.* alley side Σ_8_PAH_semivolatile_ and individual semivolatile PAHs measured across the neighborhood.

We also acknowledge that the original intent of the neighborhood-wide evaluation of outdoor PAH measurements was not to investigate differences in concentrations by sampling site. Hence, the street side and alley side measurements were not sampled simultaneously. In addition, we were unable to adequately control for variations in measurements by other major emissions sources (e.g., traffic and residential heating oil emissions), floor height, as well as influences from other environmental conditions (e.g., temperature, humidity, and wind speed), that may significantly affect neighborhood outdoor PAH concentrations. These factors may contribute some error in our measurement of PAHs. However, our two week averaged PAH measurements were exposed to various wind speeds and directions as well diurnal variations in daily traffic emissions, thus offering a comprehensive representation of exposure compared to shorter duration measurements. Yet we acknowledge that one limitation of two-week sampling is that samples are more subject to the potential degradation of nonvolatile PAHs by ambient ozone after collection on the filter as shown in our previous publication [21], likely independent of site of monitor placement. In addition, consideration of factors that contribute to the urban street canyon effect, such as distance between buildings, building setbacks, turbulence of airflow and other micrometeorological conditions should be considered in future replications studies. Despite these limitations, the crude analysis of the neighborhood-wide samples corroborates our findings at the central site and supports our main hypothesis that PAH concentrations measured at the front of urban buildings, adjacent to street traffic, are higher than those measured at the back or alley side of buildings.

## 5. Conclusions

We found that ambient PAHs, nonvolatile PAHs in particular, are significantly higher when measured from the street side of buildings compared to the alley side of buildings. Our findings raise important methodological issues in air sampling for environmental exposure research. Specifically, the location of monitor placement and the built environment is critical for accurate measurement of PAH exposure. Furthermore, this study can inform future research to identify methods of reducing ambient air pollution exposures, such as keeping street facing windows closed during high traffic periods of the day. Overall, it is important to accurately consider personal exposure to pollutants when health-related outcomes are in question.

## Abbreviations

**AM:** Arithmetic Mean
**CCCEH**: Columbia Center for Children’s Environmental Health
**GM:** Geometric Mean
**NYC**: New York City
**LOD:** Limit of Detection
**PAH**: Polycyclic Aromatic Hydrocarbons
**Σ****_8_****PAH_semivolatile_**: Sum of 8 low molecular-weight-PAH ≤ 206, including pyrene (Pye), phenanthrene (Phe), 1-methylphenanthrene (1Meph), 2-methylphenanthrene (2Meph), 3-methylphenanthrene (3Meph), 9-methylphenanthrene (9Meph), 1,7-dimethylphenanthrene (1,7DMeph), and 3,6-dimethylphenanthrene (3,6DMeph)
**Σ****_8_****PAH_nonvolatile_**: Sum of 8 high molecular-weight-PAH ≥ 228, including benz [a]anthracene (BaA), chrysene/iso-chrysene (Chry), benzo[b]fluoranthene (BbFA), benzo[k]fluoranthene (BkFA), benzo[a]pyrene (BaP), indeno[1,2,3-c,d]pyrene (IP), dibenz [a,h]anthracene (DahA), and benzo[g,h,i]perylene (BghiP).
**PUF**: Polyurethane Foam
**SD**: Standard Deviation
